# Supplementary material for: Effects of Infant Formula Type on Early Childhood Growth Outcomes: A Retrospective Cohort Study
Source: Nutrients. 2025 Sep 30;17(19):3111. doi: 10.3390/nu17193111 (PMC12525715; doi:10.3390/nu17193111)
Supplement: Supplementary file 1 [file nutrients-17-03111-s001.zip › nutrients-3859951-supplementary.pdf]

Effects of Infant Formula Type on Early Childhood Growth Outcomes: A Retrospective Cohort Study

Uzma Rani[a], MD, MPH; Roba Alwasila[b], MD, MPH; William T.Story[b], PhD; Patrick Ten Eyck [c], MS, PhD; Asher Hoberg[d]; Donna A.Santillan[d], PhD; Aamer Imdad[e], MD, MPH

Supplementary table S1: Feeding Questions from Bright Futures 2-Month Flow Sheet

"What are you Feeding your baby?" (Families can pick both breastfeeding and formula feeding)
Response options:<br>- Breastfeeding – Yes / No<br>- Formula – Yes / No<br>- Other – Yes / No
"What iron-fortified formula are you using?" (when families picked the formula option)
Response options:<br>1. Regular standard formula (e.g., Enfamil, Similac, Store brand/generic)<br>2. Premature infant formula (e.g., Neosure, Enfacare)<br>3. Hypo-allergenic formula (e.g., Alimentum, Nutramigen)<br>4. Sensitive formula (e.g., Similac Total Comfort, Enfamil Gentlease, other partially hydrolyzed formula)<br>5. Other – [Name specified]

Supplementary table S2: Comparison of eligible participants who were excluded due to missing data with the included participants.

Variable	Not Included (Missing) N=4311	Analysis Sample N=5535	p-value
Sex, N (%)			0.1435
Female	2079 (48.2%)	2752 (49.7%)	
Male	2232 (51.8%)	2783 (50.3%)	
Race, N (%)			0.4142
Black or AA	560 (13.0%)	691 (12.5%)	
Other	1036 (24.0%)	1390 (25.1%)	
White	2715 (63.0%)	3454 (62.4%)	
Insurance, N (%) [# missing = 1004]			<.0001
Medicaid	1201 (34.1%)	1834 (34.5%)	
Other	58 (1.7%)	211 (4.0%)	
Private	2262 (64.2%)	3276 (61.5%)	
Mother's Race, N (%)			0.0875
Black or AA	570 (13.2%)	718 (13.0%)	
Other	937 (21.7%)	1109 (20.0%)	
White	2804 (65.0%)	3708 (67.0%)	
Mother's Ethnicity, N (%) [# missing = 3179]			0.0171
Hispanic or Latino	193 (9.8%)	532 (11.3%)	
NOT Hispanic or Latino	1758 (89.6%)	4116 (87.5%)	
Other	12 (0.6%)	56 (1.2%)	
